# Evaluating the Antidiabetic Activity of *Aloe niebuhriana* Latex in Alloxan-Induced Diabetic Rats and the Development of a Novel Effervescent Granule-Based Delivery System

**DOI:** 10.1155/tswj/5648662

**Published:** 2025-01-09

**Authors:** Bushra Abdulkarim Moharram, Mahmoud Mahyoob Alburyhi, Tareq Al-Maqtari, Abdu Faisal

**Affiliations:** ^1^Department of Pharmacognosy, Faculty of Pharmacy, Sana'a University, Sana'a, Yemen; ^2^Department of Pharmaceutics and Industrial Pharmacy, Faculty of Pharmacy, Sana'a University, Sana'a, Yemen; ^3^Department of Pharmacology, Faculty of Pharmacy, Sana'a University, Sana'a, Yemen; ^4^Department of Microbiology, Immunology and Pharmacology, Arkansas College of Osteopathic Medicine, Arkansas Colleges of Health Education, Fort Smith, Arkansas, USA; ^5^Department of Research and Development Center, Modern Pharma Company and Global Pharmaceutical Industries, Sana'a, Yemen

**Keywords:** alloxan, *Aloe niebuhriana*, diabetes, effervescent granules

## Abstract

**Background:** Ethnomedicine exhibits potential in developing affordable effective antidiabetic agents.

**Aim:** This work aimed to explore the antidiabetic properties of *Aloe niebuhriana* latex extract both in vivo, utilizing alloxan-induced diabetic rats, and in vitro, through *α*-amylase enzyme testing. Additionally, it sought to formulate optimal effervescent granules derived from the extract.

**Methods:** The *α*-amylase inhibition assay was performed using the *α*-amylase kit using biochemical analyzers. Experimental diabetes was induced in animals with alloxan. On Day 14 postdiabetes induction, body weight, fasting blood glucose, and lipid profile parameters were determined. Also, six effervescent granule preparations of the extract were formulated using wet granulation. Based on its physical and organoleptic properties, a formulation was selected and optimized.

**Results:** The extract displayed modest *α*-amylase inhibition, with an IC_50_ value of 439.2 *μ*g/mL. Both doses of *A. niebuhriana* extract (200 and 400 mg/kg) significantly reduced blood glucose level compared to their respective Day 1 levels (*p* < 0.001). Moreover, the extract at a dose of 400 mg/kg significantly normalized lipid profile compared to the diabetic control groups (*p* < 0.05 − 0.001). Six formulations containing the extract were prepared (F1–F6), and F6 containing 200 mg of the extract was selected for optimization due to its favorable odor, taste, foaming, and effervescent properties, high solubility, and absence of turbidity and adhesion. The formulated F6 granules successfully met the quality parameters assessed including flow time, pH effervescent time, angle of repose, bulk density, tapped density, Carr's index, and Hausner's ratio.

**Conclusion:** This study highlights the antidiabetic potential of *A. niebuhriana* latex extract, potentially attributed to its hypolipidemic, hypoglycemic, and *α*-amylase inhibitory effects. The successful formulation and evaluation of the extract as effervescent granules suggest its potential as an antidiabetic drug.

## 1. Introduction

Diabetes mellitus (DM) stands as one of the most prevalent and serious chronic diseases of the current era, posing life threatening, disabling, and financially burdensome complications, and diminishing life expectancy [1]. Indeed, diabetes is considered one of the five biggest morbidities worldwide [2]. In 2021, it was estimated that 537 million people had diabetes, a figure projected to escalate to 643 million by the year 2030 and 783 million by the year 2045 [3]. Direct health expenditures due to diabetes are already close to one trillion USD and will exceed this figure by 2030. The International Diabetes Federation (IDF) Middle-East and North Africa Region has the highest percentage (24.5%) of diabetes-related deaths in people of working age [4]. Notably, type 2 diabetes accounts for over 90% of all diabetes cases [5].

While currently available antidiabetic medications are essential for managing glycemic levels, their use is often associated with significant side effects, including hypoglycemia, fluid retention, osteoporosis, and heart failure [6, 7]. These adverse effects can restrict their effectiveness in clinical practice. Consequently, there is an urgent demand for the development of novel antidiabetic therapies with fewer side effects to address diabetes and its related conditions, such as hyperglycemia, hyperinsulinemia, and hypertriglyceridemia. Natural products play a pivotal role in the development of novel medications that have enabled humanity combat several illnesses. Certain medicinal categories including antidiabetic drugs have greatly benefited from natural products [8]. Phytomedicines offer not only cost-effective solutions to diseases but also safety. Traditional herbal medicines and functional foods are believed to ameliorate diabetic symptoms through multiple mechanisms of action, including enhanced insulin secretion and sensitivity, increased glucose uptake by muscle cells and adipose tissues, inhibition of glucose absorption from the intestines, reduced glucose production by hepatocytes, and anti-inflammatory properties [9]. As a result, functional foods and phytotherapies are becoming increasingly more popular across the world day by day [10].

Nonetheless, there is a pressing need for scientific validation, standardization, and safety assessment of traditional medicinal plants before their recommendation for treating various ailments. Herbal medicine formulations, now produced by the modern pharmaceutical industry, are extensively available in the market for disease management public health enhancement [11]. The categorization of finished herbal products into specific dosage forms aids in establishing precise protocols for quality control and stability testing [12].

*Aloe niebuhriana,* belonging to the family Aloeaceae, is an indigenous species of Arabian Peninsula [13]. Similar to *Aloe* vera, a widely recognized *Aloe* species, *A. niebuhriana* has a traditional application for treating multiple ailments such as hypertension, constipation, gastrointestinal parasites, and skin diseases as well as diabetes [14]. Previous research indicates that *A. niebuhriana* contains anthraglycoside, bitter principles, alkaloid, flavonoid, saponin, coumarins, aldehydes, phenols, tannins, and phytosterols. Furthermore, *A. niebuhriana* exhibits antioxidant and antimicrobial as well as hepatoprotective activities [15].

In Yemen, medicinal plants are widely utilized for treating diseases in both humans and animals. However, only a handful of studies, often narrow in scope, have focused on documenting this indigenous knowledge [16–20]. Given Yemen's vast geographical expanse and its diverse society, culture, and ecology, these studies represent only an initial yet crucial effort toward understanding and preserving the country's traditional medicinal practices. Similar to other rural societies, Yemeni people have historically maintained a deep connection with wild plants, using them for food, medicine, cosmetics, construction materials, shelter, and clothing [21, 22]. Yemen boasts a rich diversity of plants used in traditional medicine, including many indigenous and endemic plants [16, 18–22]. Investigating the antidiabetic properties of these traditional plants offers an opportunity to uncover new pharmacological applications and potential antidiabetic agents. This study seeks to explore the antidiabetic potential of *A*. *niebuhriana* latex extract and develop the extract into an effervescent dosage form.

## 2. Materials and Methods

### 2.1. Chemicals and Reagents

Methanol (Umco, Egypt), 99.9% ethanol (Umco, Egypt), Glibenclamide (Daonil® Sanofi, Egypt), Garbose 100 mg tablets (YSP, Malaysia), 98% alloxan (Oxford laboratory, India), and bacterial *α*-amylase (BDH, UK) were employed for antidiabetic analyses. For the *α*-amylase assays, the *α*-amylase-EPS substrate (BioSystems S.A., Spain) contained two reagents: Reagent A: HEPES 50 mmol/L, calcium chloride 0.075 mmol/L, sodium chloride 90 mmol/L, magnesium chloride 13 mmol/L, *α*-glucosidase > 4 U/mL, pH 7.1 and Reagent B: HEPES 50 mmol/L, 4-nitrophenyl-maltoheptaoside-ethylidene 18 mmol/L, pH 7.1.

Excipients including anhydrous citric acid, tartaric acids, stearic acid, sodium bicarbonate, sucralose, sodium saccharin, polyvinylpyrrolidone (PVP K-30), peppermint powder (menthol), simethicone, monopropylene glycol (MPG), Aerosil® 200, tween 20, sodium lauryl sulfate, and starch were generously provided by the Modern Pharma Company and Global Pharmaceutical Industries in Sana'a, Yemen. All other used reagents in this work were of analytical grade.

### 2.2. Equipment

The following equipment was utilized for the antidiabetic assay: a centrifuge (Hermle Z400, Germany), polypropylene cages, vacuum blood collection tubes (6 mL yellow-cap tubes with gel and clot activator), and a feeding cannula. A chemistry analyzer model BS-240 (Mindray, China) was used for alpha amylase inhibition analysis. For formulation purposes, the following equipment was employed: a UV/VIS spectrophotometer (Cary 50 conc, Varian, USA), an FT-IR spectrometer (Scimitar 2000 FT-IR, Varian, USA), a PH meter (Metrohm 913, Swiss), a forced convection oven (model LDO-150F, Korea), a SCT-SIONIC-6 sonicator bath (ScichemTech, USA), a hotplate stirrer (LMS-1003, Korea), a balance (Metler), and a Stuart SMP3 melting point apparatus (Barloworld Scientific Ltd., UK).

### 2.3. Animals

Male albino rats weighing 200–250 g were housed in individual cages with regulated light and dark cycles, following a 12 h day/night schedule, and maintained under controlled ventilation, humidity, and temperature conditions (24°C ± 3°C). The animals were provided with commercial stock diet and water *ad libitum*. The stock diet was a standard laboratory formulation containing protein, fat, and carbohydrates, along with essential vitamins and minerals to meet nutritional requirements. The animals underwent a 1 week acclimatization period in the laboratory environment, during which their health status was monitored. All experimental procedures were conducted in compliance with the standard animal ethics guidelines.

### 2.4. Plant Material

The leaves of *A. niebuhriana* were harvested from Alabos village in Taiz governorate in June 2017 and subsequently authenticated at the Agricultural Research and Extension Authority (AREA) in Dhamar, Yemen. A voucher specimen of the plants was prepared and archived in the Pharmacognosy Department at the Faculty of Pharmacy, Sana'a University, with a voucher number Anieb2017.

### 2.5. Plant Extraction

The ethanol extract of *A. niebuhriana* dried latex was prepared as previously described [15]. In brief, fresh leaves of A. niebuhriana were cleaned, and latex was collected by cutting the leaves and allowing them to drain into clean glass dishes for an hour before drying. The dried latex was then extracted with ethanol and filtered. The filtrate was dried using a rotary evaporator in a water bath at a temperature below 45°C. The final dried extract was stored in desiccators for biological activity testing.

### 2.6. Antidiabetes Activity

#### 2.6.1. *α*-Amylase Inhibition (In Vitro Study)

The inhibition assay was conducted following the methodology outlined in a previous study [23] with slight modifications as described by Moharram et al. [24]. From a stock methanolic solution of *A. niebuhriana* (100 mg/mL), five serial dilutions were prepared (100, 50, 25, 12.5, and 6.25 mg/mL). Each assay mixture consisted of 40 μL of plant extract from each dilution and 560 *μ*L of methanol within Eppendorf tubes. Blank controls for each dilution of the plant extract were prepared by adding 40 μL of plant extract from each dilution to 760 μL of methanol. Serial dilutions of acarbose (5 mg/mL), serving as a positive control, were prepared in distilled water (D.W.) to achieve concentrations ranging from 1600 to 200 μg/mL. The assay mixture for acarbose consisted of 40 *μ*L of each dilution (1600-200 μg/mL) and 560 μL of D.W. mixed in Eppendorf tubes. The negative extract control, representing 100% enzyme activity, was obtained by replacing the plant extract with methanol (40 *μ*L). For the negative acarbose control, 40 *μ*L of D.W. was utilized.

The reaction commenced with the addition of 200 *μ*L of 2 mg/mL *α*-amylase solution to the previously prepared assay mixtures (extract, acarbose, and negative control) resulting in a total mixture volume of 800 μL. The tubes were then centrifuged for 3 min and incubated at room temperature for a total duration of 10 min. Subsequently, *α*-amylase-ESP was introduced into each tube as a substrate. The inhibition of *α*-amylase for each dilution was evaluated by measuring the absorbance of the liberated 4-nitrophenol at 405 nm using a chemistry analyzer. Final concentrations in the incubation mixtures were as follows: 0.313–5 mg/mL for the plant extract dilutions, 10–80 *μ*g/mL for acarbose solutions, and 0.5 mg/mL for the 20 unit/mL enzyme.

The inhibition of *α*-amylase was expressed as a percentage of inhibition and calculated using the following equation:

(1) $$\begin{array}{c} \text{inhibition }\left(\%\right)=\left(\frac{A\text{bs}.\text{ of control}-\left[\text{Abs}.\;S-\text{Abs}.\;B\right]}{\text{Abs}.\text{ of control}}\right)\times 100, \end{array}$$

where Abs. = absorbance, Abs. S = absorbance of sample, and Abs. B = absorbance of blank.

The percentage inhibition of *α*-amylase was then plotted against the sample concentration, and a logarithmic regression curve was created to calculate IC_50_ values. IC_50_ represents the concentration of the sample (*μ*g/mL) required to reduce the absorbance of *α*-amylase by 50%.

#### 2.6.2. Antidiabetic Assay (In Vivo)

Antidiabetic assay was conducted following the methodology outlined in previous studies by Moharram et al. [24] and Al-Baoqai et al. [25]. Male albino rats underwent an 18-h fasting period. Fasting blood glucose (FBG) levels were determined using blood samples from rat tail vein and analyzed with an Accu Chek Performa Advantage II auto analyzer (Roche). Hyperglycemia was induced by intraperitoneal injection of freshly prepared alloxan monohydrate dissolved in normal saline (0.9% w/v NaCl) at a single dose of 200 mg/kg. To prevent fatal hypoglycemia resulting from massive pancreatic insulin release, rats were kept on 5% glucose for the subsequent 24 h. Confirmation of hyperglycemia occurred 72 h postinjection through elevated glucose levels. Rats exhibiting blood glucose level exceeding 200 mg/dL on Day 3 postalloxan injection were categorized as diabetic rats and included in the study.

##### 2.6.2.1. Experimental Design

The antidiabetic activity of ethanolic extract was evaluated in alloxan-induced diabetic rats. Albino rats were randomly assigned to five groups, each consisting of 5 rats:

• Group 1: Normal rats without treatment, as the baseline.
• Group 2: Diabetic control rats without treatment rats.
• Group 3: Diabetic rats treated with the ethanolic extract at a dose of 200 mg/kg/day administered orally.
• Group 4: Diabetic rats treated with the ethanolic extract at a dose of 400 mg/kg/day administered orally.
• Group 5: Diabetic rats treated with glibenclamide at a dose of 5 mg/kg/day administered orally.

The treatment began on Day 3 after diabetes induction and was marked Day 1 of treatment. Prior to treatment initiation, body weights of the animals were recorded using a weighing balance, and subsequent measurements were taken on the 7^th^ and 14^th^ day of treatment. Diabetic rats receiving treatment were administered the leaf extract (dissolved in 5% Tween 80) or glibenclamide orally via gastric intubation using a force feeding needle for a duration of 2 weeks. Untreated normal and diabetic rats were provided with D.W. only. FBG levels were recorded before treatment commencement, on the seventh day and on the 14th day of treatment. Blood samples were obtained from the retro-orbital plexus on the 14th day after leaf extract administration. To collect blood samples, the rats were anaesthetized with diethyl ether and euthanized via cervical dislocation. Blood was collected into vacuum blood collection tubes containing the biochemistry serum. The samples were allowed to clot, and serum was separated by centrifugation at 3000 g for 15 min before being analyzed for various biochemical parameters.

##### 2.6.2.2. Assessment of Serum Lipid Profile Levels in Experimental Animals

Serum lipid profile, including triglycerides (TG), total cholesterol (TC), high-density lipoprotein (HDL), and low-density lipoprotein (LDL), was determined colorimetrically using assay kit method from Mindray (China diagnostics) following the manufacturer's instructions. The assays were conducted at Sehatak Laboratories for Medical Analysis in Sana'a Yemen.

### 2.7. Formulation of Effervescent Granules

The effervescent granules of *A. niebuhriana* latex extract were prepared using the wet granulation method, as previously outlined by Al-Mousawy et al. [26] with minor adjustments.  Table 1 details the composition and quantities of each ingredient employed. Following the principles of geometrical dilution, all components were thoroughly mixed to ensure uniform dispersion of the extract. The resulting powder was then sieved through mesh no. 25. After that, a suitable amount of granulating agent (ethanol 99.9%) was added to form a moist mass. This moist mass was passed through sieve no. 16 to obtain granules. The granules formed were dried overnight in a hot air oven at 40°C and sealed in an airtight container.

### 2.8. Evaluation of Formulated Effervescent Granules

#### 2.8.1. Evaluation of the Organoleptic and Physical Properties

The six effervescent formulations underwent assessment for their organoleptic properties, encompassing attributes such as shape, color, aroma, and taste. In addition, the physical properties of the granules, including foaming, adhesion, effervescent, turbidity, and solubility, were also evaluated.

#### 2.8.2. Flowability Study

The flowability test was conducted only on the formulation that exhibited satisfactory results in the organoleptic and physical tests. Employing the funnel method, the flow rate and angle of repose were measured. A funnel was positioned on either a ring support stand or a lambed stand, adhering to pharmacopeial standards for height. Approximately, 10 g of the granule sample was placed in the funnel with the bottom hole closed. Subsequently, the lower lid of the funnel is opened to allow the granules to descend onto a level surface, forming a conical pile. The flow time duration from the initiation of granule flow until cessation was recorded using a stopwatch. The flow rate of the sample and the angle of repose of the sample were calculated as follows:

(2) $$\begin{array}{c} \text{flow rate}=\frac{\text{weight }\left(\text{grams}\right)}{\text{time }\left(\text{seconds}\right)}. \end{array}$$

The angle of repose was calculated using the following formula:

(3) $$\begin{array}{c} \text{Tan}\theta =\frac{h}{r}, \end{array}$$

where *θ* represents the angle of repose, h represents the height of the formed pile cone, and r represents the radius of the base of the cone [26, 27].

#### 2.8.3. Bulk Density (BD) and Tapped Density (TD)

Two types of density, namely, BD and TD, were determined following the procedures outlined in Kevin and Aulton [28]. A measured quantity of granules was placed into a measuring cylinder, and the initial volume was recorded to determine the BD. Subsequently, the measuring cylinder was subjected to tapping at a rate of 100 taps per minute until maximum packing was achieved. The powder level in the measuring cylinder was checked after every 10 taps until no further reduction in volume occurred. BD and TD were then calculated using the following formulas:

(4) $$\begin{array}{c} \text{BD}=\frac{\text{granules weight}}{\text{packing volume}}, \end{array}$$

(5) $$\begin{array}{c} \text{TD}=\frac{\text{granules weight}}{\text{tapped volume of packing}}. \end{array}$$

To assess the flow properties and compressibility of the effervescent granules derived from *A. niebuhriana* extract, Carr's Index (CI) and Hausner Ratio (HR) were determined, following the methodology described by Kevin and Aulton [28]. These were calculated using the equations:

(6) $$\begin{array}{c} \text{CI}=\left(\frac{\text{TD}-\text{BD}}{\text{TD}}\right)\times 100, \end{array}$$

(7) $$\begin{array}{c} \text{HR}=\frac{\text{TD}}{\text{BD}}. \end{array}$$

#### 2.8.4. Effervescence Time

The effervescent time for the granules was determined by introducing a single dose (2.5 g) of the *A. niebuhriana* extract effervescent granules into a glass containing 200 mL of water, and the duration time until effervescent cessation was recorded [29].

#### 2.8.5. PH Test

For the pH test, the effervescent solution was prepared by dissolving 2.5 g of the granules in 100 mL of water, followed by pH measurement using a pH meter. A pH reading within the range of 6 to 7 is considered optimal for the effervescent solution, indicating neutrality [30].

### 2.9. Packing of the Finished Product

The optimized effervescent granules formulation was prepared and packed.

### 2.10. Statistical Analysis

For statistical analysis, one-way analysis of variance (ANOVA) was employed. Data were represented as mean ± standard error of the mean (mean ± SEM). Tukey's multiple comparison test was utilized to compare mean values across different groups treated with the extract and the control groups. A significance level of *p* < 0.05 was considered statistically significant.

## 3. Results

### 3.1. In Vitro *α*-Amylase Inhibition Activity

**Figure 1:**
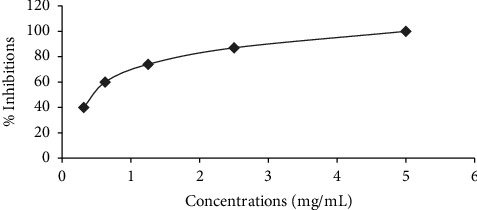
The percentage inhibition of *α*-amylase by *A. niebuhriana* latex extract at different concentrations. Each data point represents the mean of triplicates, and the vertical bars indicate the standard deviations of mean.

**Figure 2:**
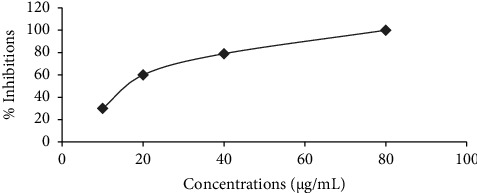
The percentage inhibition of *α*-amylase by acarbose (a positive control) across various concentrations. Each data point represents the mean value from triplicates, with the standard deviations of the mean represented by vertical bars.

**Figure 3:**
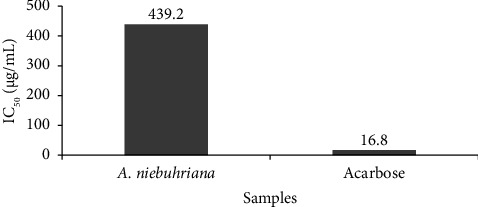
IC_50_ (μg/mL) for the *α* amylase inhibitory activity of *A. niebuhriana* latex extract and for acarbose.

Figures 1 and 2 illustrate the *α*-amylase inhibitory activity of *A. niebuhriana* latex extract alongside acarbose, serving as a positive control. The observed effects demonstrated a dose dependent effect, as the inhibitory activity intensified with escalating concentrations. The extract exhibited 100 ± 0.5% inhibition of *α*-amylase at a concentration of 5 mg/mL. The IC_50_ values were determined, as depicted in Figure 3. The *A. niebuhriana* latex extract displayed modest *α*-amylase inhibition, with an IC_50_ value of 439.2 *μ*g/mL. However, it is noteworthy that the inhibition potency was comparatively lower than that of acarbose, which exhibited an IC_50_ value of 16.8 μg/mL.

### 3.2. In Vivo Antidiabetic Activity

#### 3.2.1. Effect of *A. niebuhriana* Latex Extract on Body Weight of Diabetic Animals

**Figure 4:**
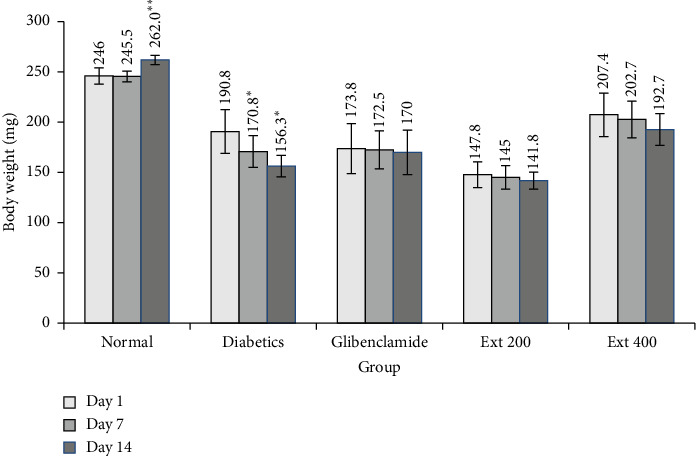
Mean body weight changes in alloxan-induced diabetic rats following a 14-day treatment regimen with *A. niebuhriana* extract at doses of 200 (ext 200) and 400 (ext 400) mg/kg, as well as glibenclamide (5 mg/kg). The results are expressed as the mean ± SEM (*n* = 5), with standard error of the mean (SEM) indicated as vertical bars. Values with ⁣^∗^*p* < 0.05 and ⁣^∗∗^*p* < 0.01 were considered significant compared to the respective day 1 measurements. A one-way ANOVA followed by Tukey's multiple comparison test was used to compare the groups.

In Figure 4, the impact of *A. niebuhriana* and glibenclamide on alterations in body weight is depicted. Notable, the weight of the normal group exhibited a significant increase (*p* < 0.05) on Day 14 (262.0 ± 4.6 mg) compared to Day 1 (246 ± 8.2 mg). Conversely, in diabetic control rats, a significant decrease (*p* < 0.05) in final body weight on Days 7 (170.8 ± 15.8 mg) and 14 in comparison to Day 1 (190.8 ± 10.8 mg). However, treatment with *A. niebuhriana* and glibenclamide did not result in any significant (*p* > 0.05) change in the body weight of diabetic rats.

#### 3.2.2. The Impact of *A. niebuhriana* on Blood Glucose in Experimental Groups

Table 2 displays the effect of *A. niebuhriana* latex extract on the blood glucose levels of experimental animals was determined at Days 1, 7 and 14 post oral administrations. A significant increase in blood glucose was observed in alloxan-induced diabetic rat (*p* < 0.001), compared to the normal group. However, administration of the extract at both doses (200 and 400 mg/kg) and glibenclamide resulted in a significant reduction in blood glucose concentration by Days 7 and 14.

The crude extract administered at a daily dose of 200 mg/kg significantly reduced FBG levels on Day 7 (*p* < 0.01) and Day 14 (*p* < 0.001) compared to the respective levels on Day 1. Similarly, administration of the extract at a daily dose of 400 mg/kg led to a significant decrease in the FBS level significantly (*p* < 0.001) on Days 7 and 14. Furthermore, significant reductions in FBS levels were also observed with the standard drug glibenclamide, on Days 7 and 14 (*p* < 0.01) compared to the respective levels on Day 1. Notably, on Day 14, the higher dose of 400 mg/kg of the latex extract significantly outperformed glibenclamide in lowering FBG levels, suggesting a remarkably potent antidiabetic activity. Meanwhile, the FBG-lowering effect of the lower 200 mg/kg dose was not statistically different from that of glibenclamide, suggesting it may be similarly effective at this concentration but not as potent as the higher dose. The observed effects suggest dose and time-dependent activity, as diabetic rats treated with the 400 mg/kg extract on Days 7 and 14 displayed a higher decrease in FBG levels (62.2% and 78%, respectively) compared to those treated with the lower 200 mg/kg dose (29.2% and 73.1%, respectively).

#### 3.2.3. Effect of *A. niebuhriana* on Serum Lipid Profile

Table 3 presents the serum levels of TG, TC, LDL, and HDL of rats in the experimental groups. Rats in the diabetic group (alloxan group) exhibited a significant increase (*p* < 0.01–0.001) in TC, TG and LDL levels, along with a decrease in HDL levels in comparison with the normal group. Treatment with glibenclamide and *A. niebuhriana* extract at a dose of 400 mg/kg resulted in a significant decrease in serum TC, TG and LDL levels, along with a significant increase in HDL levels after 14 days of treatment compared to the diabetic group. However, treatment with A. niebuhriana at a lower dose (200 mg/kg) led to a significant increase only in TC and LDL levels (*p* < 0.01) compared to the diabetic control rats.

### 3.3. Formulation of *A. niebuhriana* Effervescent Granules

In this study, *A. niebuhriana* latex extract exhibited potent antidiabetic activity in both in vivo and in vitro experiments. Consequently, the extract was formulated into effervescent granules. Six formulations were prepared using the wet granulation method, each containing aa combination of various ingredients listed in Table 1. The ingredients across the six formulations were included different amounts of the extract, citric acid, tartaric acids, stearic acid, sodium bicarbonate, sucralose, sodium saccharin, PVP K-30, peppermint powder (menthol), simethicone, MPG, aerosil® 200, tween 20, sodium lauryl sulfate, and starch.

**Figure 5:**
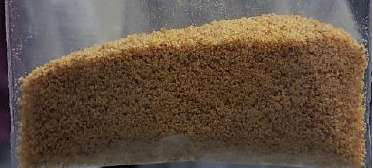
An image of the formulated effervescent granules from *A. niebuhirana* extract.

Table 4 presents the organoleptic and other physical properties of the six prepared formulations. The properties evaluated included foaming, adhesion, effervescence, turbidity, floating and solubility. Based on these properties, formulation 6 (F6) was selected for further optimization due to favorable qualities such as odor, taste, foaming, high solubility properties with no formation of apparent turbidity or adhesion.  Figure 5 displays the formulated effervescent granule (F6). This chosen formulation (F6) contained the following components: extract (200 mg), Na bicarbonate (833.3 mg), citric acid (383.3 mg), tartaric acid (695.0 mg), sucralose (50 mg), peppermint (16.7 mg), tween 20 (30.0 mg), Na saccharin (83.3 mg), aerosil (50.0 mg), simethicone (0.3 g), MPG (0.3 g), and PVP K30 (0.15 g).

### 3.4. Evaluation of the *A. niebuhriana* Effervescent Granules

Table 5 summarizes the evaluation of F6 for its pH, effervescent time, flow time, angle of repose, BD, TD, Carr's index, and Hausner's ratio. The granules were observed to be yellow in color, with a pleasant peppermint odor and taste. The results of the evaluation revealed the following parameters for the formulated effervescent granules: pH of 5.28, angle of repose of 24.4°, BD of 0.62 g/mL, TD of 0.72 g/mL, Carr's index of 14.29, Hausner ratio of 1.17, and effervescent time of 60 s.

## 4. Discussion

*A. niebuhriana* is an endemic species of Arabian Peninsula and in Yemen [13]. Traditionally, *A. niebuhriana* is utilized in a manner akin to *Aloe vera*, a commonly known *Aloe* species, for treating various ailments, including diabetes [31].

In contemporary medicine, managing diabetes without incurring severe side effects presents a considerable challenge, given the potential adverse effects associated with many antidiabetic drugs [6, 7]. Consequently, there is a growing preference for medicinal plants with promising antidiabetic properties due to their perceived lower incidence of side effects and cost-effectiveness [32].

This study represents the first documentation of the *α*-amylase inhibitory, antidiabetic, and hypolipidemic effects of *A. niebuhriana* latex extract, shedding light on its potential as a natural alternative for managing diabetes with reduced risk of adverse effects.

In this study, *A. niebuhriana* extract displayed modest inhibition of *α*-amylase activity. One approach to managing type-2 diabetes is to reduce post-prandial hyperglycemia, a condition linked to oxidative stress and endothelial dysfunction, both of which play a role in diabetic complications. This can be accomplished by inhibiting glucose absorption through the suppression of the carbohydrate-digesting enzymes in the gastrointestinal tract, specifically pancreatic *α*-amylase and intestinal *α*-glucosidase [33]. Acarbose, along with miglitol and voglibose, are used as therapeutic agents for diabetes via inhibiting these enzymes [34]. In this study, *A. niebuhriana* extract at a concentration of 5 mg/mL exhibited 100% *α*-amylase inhibition outperforming *Aloe vera*, a well-known Aloe species, which showed 87% inhibition [35]. The variation in enzyme inhibition potency between *A. niebuhriana* and other Aloe species may be due to differences in phytochemical composition. Several studies have shown that Aloe species can have hypoglycemic effects by effectively inhibiting pancreatic *α*-amylase activity [35–37]. These studies along with the findings of the current study may exert hypoglycemic effects through the inhibition of pancreatic *α*-amylase, potentially contributing to its antidiabetic properties.

The study also assessed the hypoglycemic and hypolipidemic effects of *A. niebuhriana* latex extract in alloxan-induced diabetic rats. Alloxan, a toxic diabetogenic compound, is commonly used to induce diabetes in experimental animals. Alloxan exerts its diabetogenic effect by generating superoxide radicals, leading to cellular damage and a subsequent increase in cytosolic calcium concentration, ultimately resulting in the destruction of pancreatic beta cells and consequent hyperglycemia [38]. Alloxan administration at doses ranging from 150 to 200 mg/kg intraperitoneally has been reported to elevate blood glucose levels and cause damage to pancreatic *β* cells in mice. Hyperglycemia in mice or rats is typically defined by blood glucose levels exceeding 200 mg/dL [24, 39, 40]. In the present study, a significant increase in glucose levels was observed in the diabetic animals following alloxan administration. However, oral administration of the aqueous extract of A. niebuhriana significantly lowered serum glucose in those animals.

In our previous study, we found that the *A. niebuhriana* latex extract did not cause any observable toxic effects in rats when administered at a high dose of 4000 mg/kg. This indicates that the extract is well tolerated by rats, as no adverse effects were observed during the observation period [41]. Therefore, to conduct the subsequent antidiabetic testing, doses of 200 and 400 mg/kg body weight, which represent one-tenth [42] of the highest dose, were selected in the current study. The selected doses are within a reasonable range that reflects both its traditional application and the expected therapeutic window, ensuring a balance between efficacy and safety. Based on the results of the present study, *A. niebuhriana* latex extract administered at both doses of 200 and 400 mg/kg body weight induced a significant reduction in FBG levels on Days 7 and 14, compared to baseline levels (before treatment). Importantly, the antidiabetic effect of *A. niebuhriana* was found to be dose-dependent, with the 400 mg/kg body weight dose exhibiting a more pronounced effect than the 200 mg/kg body weight dose. This finding is consistent with previous studies demonstrating the blood glucose-lowering effects of *A. vera*, a closely related Aloe species, in alloxan-induced diabetic animals [43, 44].

Notably, treatment with *A. niebuhriana* latex extract at a dose of 400 mg/kg resulted in a significant reduction of 78% in FBG levels after 14 days, surpassing the effects observed with glibenclamide, a standard oral hypoglycemic medication. This enhanced action may be attributed to the ability of A. niebuhriana extract to potentiate pancreatic insulin secretion from *β*-cells within the islets, similar to the mechanism of action of glibenclamide. Additionally, *A. niebuhriana* extract may exert protective effects on *β*-cell viability, potentially preventing *β*-cell death or promoting the recovery and proliferation of partially damaged *β*-cells, as observed with other Aloe species [45]. The observed *α*-amylase inhibition and antidiabetic effects of *A. niebuhriana*'*s* latex shown in this study suggests the involvement of phytochemicals reported in the Aloe plant genus such as glycosides, flavonoids, tannins, alkaloids, saponins and phenols [15, 46–48]. The specific compounds responsible for the antidiabetic activity of *A. niebuhriana* latex are not yet identified. However, various anthraquinones and polyphenols have been found in Aloe species with barbaloin, aloeresin and aloe emodine being the most notable [49]. Aloeresin A, isolated from *A. ferox* has shown inhibitory effects on both maltase and sucrase [50], while chrysalodin, isolated from A. vera, strongly inhibits *α*-glucosidase [51]. In addition, anthraquinones such as aloe-emodin, emodin and chrysophanol are known to possess antiglycation properties [52]. A research has also indicated that the inhibition of *α*-amylase and *α*-glucosidase by Aloe vera leaf extract is attributed to flavonoids, which may help reduce postprandial hyperglycemia and contribute to its anti-diabetic effects [53]. The reduction in body weight observed in the diabetic animals following a 14-day following alloxan administration could be attributed to enhanced muscle protein wastage, both of which are common symptoms of insulin deficiency. Destruction of *β*-cells typically accompanies this, leading to reduced body weight and increased food and water intake—a hallmark of diabetes due to metabolic alterations stemming from insulin deficiency [54]. However, the administration of *A. niebuhriana* latex extract in this study aided in restoring rat weight to normal levels. This effect may be linked to the presence of antioxidant biochemicals like phenols and saponin, known for their potential to regulate weight [39].

The alteration of serum lipid profile, a known occurrence in alloxan-induced diabetes rats [55], was confirmed in the present study. Diabetic control rats exhibited significantly elevated TC, TG and LDL levels alongside reduced HDL compared to normal rats. Such alterations in serum lipid may potentially raise the risk of coronary heart disease [56]. In our investigation, the administration of 400 mg/kg of *A. niebuhriana* latex extract notably reduced TC, TG and LDL levels while increasing HDL. Meanwhile, the lower dose of the extract (200 mg/kg) significantly affected only the increase in the TC and LDL. This indicates that treatment with the extract restored the serum lipid profile in a dose-dependent manner. These results are in agreement with a previous report, where daily intake of the *Opuntia dillenii* seed oil significantly reduced TC, HDL, TG, and the atherogenic index levels, without affecting HDL [57]. The beneficial effect of *A. niebuhriana* latex extract (400 mg/kg) may be attributed to the extract's potential to enhance pancreatic secretion of insulin from *β*-cells in Langerhans islets, thereby enabling insulin to activate lipoprotein lipase and enhance lipid breakdown [56]. These findings are consistent with prior research reporting the hypolipidemic effect of *A. vera* [58, 59]. The lipid-lowering activity of *A. niebuhriana* may stem from the presence of polysaccharides, as identified in our previous research [15, 59]. Interestingly, in the realm of antilipidemic agents, the coating of the abdominal wall has emerged as a primary mechanism of action for gelling polysaccharides. This is owing to the gelation process, which impedes enzyme mobilization crucial for starch hydrolysis and glucose absorption [60, 61].

Per the World Health Organization (WHO), over 60% of the global populations continue to depend on herbal medicine to address both short-term and chronic ailments. Owing to extensive usage, numerous pharmaceutical companies have ventured into producing diverse herbal formulations [62]. The growing acceptance of herbal formulations is attributed to their affordability, efficacy, and lower toxicity profile. Ensuring the effectiveness, therapeutic efficacy, quality, and adherence to product standards are important considerations for any herbal formulation [63–65]. In the present study, *A. niebuhriana* latex extract was formulated and optimized as effervescent granules. Effervescent granules, comprising a mix of acids and bases, exhibit a unique property: when dissolved in water, they generate foam and mimic the taste of soft drinks [66]. Effervescence is known to enhance the dissolution and aids in masking the unpleasant taste and offer a refreshing experience when consumed [67, 68].

In our study, six effervescent formulations were prepared containing 200 or 400 mg of the plant extract, since these two doses were shown effective to manage diabetic symptoms in rats. The goal was to produce effervescent granules utilizing a combination of citric acid and tartaric acid as acid-forming effervescent salts. This combination yielded granules of superior quality, characterized by a neither nonsticky and nonbrittle texture [69]. For the base-forming effervescent salt component, we employed sodium bicarbonate, which proved to generate a substantial amount of carbon dioxide (CO2) during the effervescent reaction. These measures have proved effective in heightened user preference [70]. Tween 20 and SLS served as dispersing and wetting agents in our formulation. Notably, Tween 20 exhibited lower foaming tendencies compared to SLS. Sucralose and sodium saccharin were used as sweeteners, while peppermint powder, Aerosil 200, simethicone, povidone, and MPG were added to enhance flavor, improve powder flow, mitigate foam formation, act as a binder, and assist in wetting, respectively [71].

To ensure uniformity and homogeneity of the powder mixture, the blend of extract powder and excipients underwent sieving using a mesh size of 25 to break up powder clumps [72]. Absolute ethanol (99.9%) served as a granulating agent due to its nonactivation of the effervescent reaction and its easy removal from the wet granules at low temperatures [73]. After that, the dried granules were sieved through a mesh size of 16 to yield identical granules characterized by flow well properties and minimal weight variation when filled [72].

The flow time of 100 g of granules ≤ 10 s, as observed in our investigation, indicates a favorable flow rate [30], as demonstrated by the flow rat test. Moreover, an angle of repose falling within the range of 20°–30° indicates favorable flow properties, while angles of repose exceeding 40° suggest poor flow properties [26]. In this study, the formulated extract granules had an angle of repose 24.4°, indicating good flow properties. Furthermore, a Hausner ratio serves as another indicator of flow properties, with values below 1.25 indicative of good flow and values above 1.6 suggesting poor flow [26, 27]. Our prepared formulation had a Hausner ratio of 1.17, confirming the granules favorable flow properties.

Adjusting the pH of the effervescent solution is crucial to minimize gastrointestinal irritation. Overly acidic effervescent solutions can potentially irritate the stomach [74]. Conversely, alkaline solution may impart a bitter taste. The pH value of the selected formulation (F6) was 5.28, which falls within the desirable range close to neutral [30]. Effervescence time, on the other hand, refers to duration it takes the granules to fully dissolve into solution and produce gas. In the current study, the value of effervescence time of the selected formulation (F6) was 60 s, which falls within the acceptable range according to USP [75].

The granules of F6 exhibited a distinctive yellow color and emitted a characteristic mint odor and taste. To preserve the quality of these granules, they were packaged into special sachets comprising four layers: polyvinyl, aluminum, paper, and polyvinyl. These sachets offer impermeability to moisture and possess weak heat conduction properties, thereby safeguarding the integrity of the product.

The study demonstrates strengths in its comprehensive approach, assessing both in vitro and in vivo antidiabetic effects of *A. niebuhriana* latex and innovatively developing an effervescent granule formulation to improve practicality and user compliance. Its rigorous methodology and detailed formulation analysis enhance replicability, while the focus on affordability and safety aligns with the needs of resource-limited settings. However, there are some limitations that could limit the results of this study such as sample sizes and lack of comparison with other standard antidiabetic drugs such as metformin. In addition, the absence of long-term toxicity studies may restrict the immediate applicability of the results to clinical settings.

## 5. Conclusion

The study demonstrates that *A. niebuhriana* latex extract possesses moderate *α*-amylase inhibitory activity, with both dosages tested (200 and 400 mg/kg) showing significant hypoglycemic and hypolipidemic effects. Notably, the 400 mg/kg dose exhibited more pronounced antidiabetic properties, particularly by the seventh day of treatment, indicating a dose-dependent efficacy. These findings substantiate the traditional use of *A. niebuhriana* in promoting health and managing diabetes and its complications. Additionally, the successful formulation of *A. niebuhriana* latex extract as effervescent granules via the wet granulation method highlights the potential for its pharmaceutical application. The optimized formulation (F6), comprising *A. niebuhriana* extract, sodium bicarbonate, citric acid, tartaric acid, sucralose, peppermint, Tween 20, sodium saccharin, Aerosil, simethicone, MPG, and PVP K30, provides a promising candidate for further clinical evaluation of its antidiabetic efficacy. Continued research on the clinical efficacy and safety of F6 effervescent granules is essential to support their potential role as a therapeutic option in diabetes management.

## Figures and Tables

**Table 1:** The formulation used to prepare the effervescent granule from *Aloe niebuhriana* extract.

Ingredients (mg)	F1	F2	F3	F4	F5	F6
Extract	400.0	400.0	400.0	200.0	200.0	200.0
Sodium bicarbonate	960.0	1750.0	1750.0	866.7	883.3	833.3
Citric acid	430.0	833.3	766.7	366.7	333.3	383.3
Tartaric acid	870.0	1666.7	1500.0	746.7	703.3	695.0
Sucralose	20.0	100.0	100.0	50.0	50.0	50.0
Sodium lauryl sulfate	6.0	—	—	—	—	
Starch	300.0	—	—	—	—	
Peppermint	14.0	23.3	23.3	10.0	16.7	16.7
Tween 20	—	60.0	166.7	—	30.0	30.0
Na saccharin	—	166.7	166.7	80.0	83.3	83.3
Ascorbic acid	—	—	26.7	—	—	
Aerosil	—	—	100.0	80.0	50.0	83.3
Simethicone	—	—	—	50.0	50.0	50.0
Stearic acid	—	—	—	50.0	—	—
Monopropylene glycol	—	—	—	—	50.0	50.0
PVP K-30	—	—	—	—	50.0	25.0
Total (mg)/sachet	3000	5000	5000	2500	2500	2500

**Table 2:** Effects of *A. niebuhriana* latex extract on blood glucose levels of alloxan-induced diabetic rats after a 14-day treatment regimen.

Groups	Blood glucose levels (mg/dL) (% inhibitions)		
	Day 1	Day 7	Day 14
Normal	105.5 ± 13.0	87.5 ± 4.6	96.8 ± 9.2
Diabetic control	594.8 ± 5.3^a^⁣^∗∗^	495.5 ± 24.6^a^⁣^∗∗^	389.3.0 ± 12.0^a^⁣^∗∗^
Glibenclamide	579.3 ± 23.2^a^⁣^∗∗^	198.3 ± 26.3^b^⁣^∗∗^ (65.8)	146.5 ± 18.8^b^⁣^∗∗^ (74.0)
*A. niebuhriana* extract (200 mg/kg)	430.1 ± 20.8^a^⁣^∗∗^	304.3 ± 10.5^b^⁣^∗^^c^⁣^∗^ (29.2)	115.8 ± 10.4^b^⁣^∗∗^ (73.1)
*A. niebuhriana* extract (400 mg/kg)	450.3 ± 20.0^a^⁣^∗∗^	170.3 ± 22.7^b^⁣^∗∗^ (62.2)	99.7 ± 2.7^b^⁣^∗∗^^c^⁣^∗^ (78.0)

*Note:* Data expressed as the mean ± SEM (*n* = 5). A one-way ANOVA followed by Tukey's multiple comparison test was used to compare the groups.

^a^Significance compared to respective normal group.

^b^Significance compared to respective values on Day 1.

^c^Significance compared to the positive control group (glibenclamide-treated).

⁣^∗^*p* < 0.01 and ⁣^∗∗^*p* < 0.001 were considered significant.

**Table 3:** Effect of *A. niebuhriana* latex extract on serum lipid profile parameters in alloxan-induced diabetic rats after 14 days of treatment.

Groups	Lipid profile (mg/dL)			
	TC	TG	LDL	HDL
Normal	62.75 ± 3.3	38.0 ± 2.3	20.5 ± 2.2	52.3 ± 3.1
Diabetic control	88.8 ± 3.8^a^⁣^∗∗∗^	50.3 ± 5.0^a^⁣^∗∗^	32.3 ± 1.3^a^⁣^∗∗^	40.0 ± 2.1^a^⁣^∗∗^
Glibenclamide	52.1 ± 5.4^b^⁣^∗∗^	12.5 ± 1.0^b^⁣^∗∗^	22.3 ± 1.4^b^⁣^∗∗∗^	47.3 ± 1.6^b^⁣^∗^
Extract (200 mg/kg)	57.5 ± 5.0^b^⁣^∗∗^	48.3 ± 1.8	15.8 ± 2.4^b^⁣^∗∗^	45.5 ± 2.9
Extract (400 mg/kg)	63.3 ± 3.9^b^⁣^∗∗^	45.2 ± 4.5^b^⁣^∗^	21.5 ± 4.8^b^⁣^∗^	48.8 ± 3.3^b^⁣^∗∗^

*Note:* Data expressed as means ± SEMs (*n* = 5). A one-way ANOVA followed by Tukey's multiple comparison test was used to compare the groups.

Abbreviations: HDL: high-density lipoprotein, LDL: low-density lipoprotein, TC: total cholesterol, TG: triglycerides.

^a^Significant compared to respective normal group.

^b^Significant compared to respective diabetic control group.

⁣^∗^*p* < 0.05, ⁣^∗∗^*p* < 0.01, and ⁣^∗∗∗^*p* < 0.001 were considered significant.

**Table 4:** The organoleptic and physical properties of the six *Aloe niebuhriana* formulations.

Tests	F1	F5	F7	F12	F16	F20
Color	Yellow	Yellow	Yellow	Yellow	Yellow	Yellow
Odor	Moderate	Moderate	Good	Good	Good	Good
Taste	Bad	Good	Good	Good	Good	Good
Foaming	High	High	High	Moderate	Good	Good
Adhesion	+	+	−	+	+	−
Effervescence	Good	High	Good	Good	Good	Good
Turbidity	High	Clear	Clear	Clear	Clear	Clear
Solubility	Poor	Poor	Moderate	Moderate	High	High

**Table 5:** Physical characteristics of formulation 6 (F6) effervescent granules.

Physical characteristic	Result
Flow time (g/sec)	10.0
Angle of repose	24.4°
Bulk density (g/mL)	0.62
Tapped density (g/mL)	0.72
Carr's index	14.29
Hausner ration	1.17
pH	5.28
Effervescent time (sec)	60.0
Color	Yellow
Odor	Peppermint
Taste	Peppermint

## Data Availability

All data that support the findings of this study are available and presented in this manuscript.
